# The effects of the new configurations to invasive device associated infections in our intensİve care unit

**DOI:** 10.1186/2197-425X-3-S1-A1018

**Published:** 2015-10-01

**Authors:** M Kaynar, İ Erayman, F Gök, A Yosunkaya

**Affiliations:** Necmettin Erbakan University Meram Medical Faculty, Department of Anesthesiology and Intensive Care, Konya, Turkey; Necmettin Erbakan University Meram Medical Faculty, Department of Infectious Diseases, Konya, Turkey; Necmettin Erbakan University, Meram Medical School, Department of Anesthesiology and Intensive Care, Konya, Turkey

## Objectives

In our study we aimed to retrospectively investigate the effects of the new configuration in our ICU based on ICU design standards of The Ministry of Health of Turkey on invasive device associated infections

## Methods

A total of 526 patients, who were admitted to the ICU for more than 48 hours between 2007-2011 have been included in this study. 159 patients who have been admitted before the new configuration between 2007-2008 created Group I and 367 patients who have been admitted after the new configuration between 2009-2011 created Group II. Demographic characteristics, reasons for hospitalisation, APACHE II and Glasgow Coma Score (GCS) of all patients included in the study were recorded.Device associated infections rate which was calculated as the number of infections developed per 1000 device days,invasive procedures,device utilisation ratio,infection rates were determined.Calculations and diagnosis were made according to Centers for Disease Control and Prevention (CDC) criteria.

## Results

The difference between two groups in terms of demographic characteristics, length of stay and APACHE II scores (p > 0.05) was not statistically significant, while in Group II GCS significantly higher (p < 0.05). The most common cause of admission into ICU in both groups were found to be trauma . In group I, infection rates associated with invasive device were ventilator-associated pneumonia was 58% (VAP), catheter-associated urinary tract infection was 21.6% (CAUTI), and central venous catheter-related bloodstream infection was 20.4% (CVCR-BSI), while in Group II infection rates were VAP in 67.4%, CAUTI in 13.5% and CVCR-BSI in 19.1%. Device associated infections developed per 1000 days ) was statistically significantly higher in Group I to Group II (22:21 vs. 8.62) (p < 0.05). The ventilator utilisation ratio , central venous catheter utilisation ratio and urinary catheter utilisation ratio was similar in both groups (p > 0.05), while the VAP rate, CVCR-BSI rate, CAUTI rate was significantly lower in Group II according to Group I (p < 0.05). In both groups, the most frequently isolated device associated infections agent was Acinetobacter baumannii.

## Conclusions

As a result of our study, it shows that improving the physical conditions of the ICU reduce invasive device associated infections in ICUs.

